# Could Xuebijing Injection Reduce the Mortality of Severe Pneumonia Patients? A Systematic Review and Meta-Analysis

**DOI:** 10.1155/2020/9605793

**Published:** 2020-08-28

**Authors:** Juan Wang, Jia Zhu, Jie Guo, Qian Wang

**Affiliations:** ^1^Department of Intensive Medicine, Nanjing Hospital of Chinese Medicine Affiliated to Nanjing University of Chinese Medicine, Nanjing 210012, China; ^2^Postgraduate College, Nanjing University of Chinese Medicine, Nanjing 210023, China; ^3^Department of Respiratory Medicine, Jiangsu Province Hospital of Chinese Medicine, Affiliated Hospital of Nanjing University of Chinese Medicine, Nanjing 210029, China; ^4^Department of Chinese Medicine, Sir Run Run Hospital, Nanjing Medical University, Nanjing 211100, China

## Abstract

**Methods:**

Databases including PubMed, Cochrane Library, Web of Science, Embase, CNKI, WanFang, and VIP were searched, from inception to February 2020, to identify randomized controlled trials (RCTs) about XBJ combined with western medicine treatment in treating severe pneumonia. Literature screening, data extraction, and methodological quality assessment were carried out by two researchers back-to-back. RevMan 5.3 software was used for statistical analysis.

**Results:**

A total of 21 articles involving 2072 patients were included. The meta-analysis showed that treatment combined with XBJ has better efficiency compared with western medicine treatment alone. It could also decrease 28-day mortality; shorten the length of intensive care unit (ICU) stay time and mechanical ventilation time; and reduce the levels of C-reactive protein (CRP), procalcitonin (PCT), white blood cell (WBC), tumor necrosis factor-*α* (TNF-*α*), interleukin-6 (IL-6), and D-dimer in the serum of patients. The incidence of adverse reactions did not increase significantly.

**Conclusion:**

XBJ combined with western medicine treatment has significant clinical efficacy and no obvious adverse reactions. A dose of 100 ml bid is recommended to reduce 28-day mortality. The conclusion needs to be further verified with larger-sample size and higher-quality RCTs.

## 1. Introduction

Severe pneumonia is mainly manifested as respiratory failure, usually accompanied by systemic inflammation, which can occur with septic shock, multiple organ failure, diffuse intravascular coagulation, and so on. Severe pneumonia has become one of the main causes of death in hospitalized patients in intensive care unit (ICU), with long hospital stay and high mortality [1–3]. At present, the conventional western medicine treatment mainly involves antibiotics, mechanical ventilation, vasoactive drugs, nutritional support, and so on. However, there is no specific drug [4]. The treatments mentioned above cannot curb the progress of the body's inflammatory storm, which may be one of the reasons for the high mortality rate of patients with severe pneumonia.

In recent years, Chinese medical workers have used Xuebijing injection (XBJ) with the functions of promoting blood circulation and removing blood stasis to treat severe pneumonia and have achieved good clinical results. In the treatment of COVID-19, XBJ is one of the most frequently used traditional Chinese medicine preparations in China, and it has shown significant effects on severe patients.

Systematic reviews of XBJ in the treatment of severe pneumonia were published in 2012, 2014, and 2015, respectively, in Chinese journals [5–7], all of which confirmed the effectiveness of XBJ in the treatment of severe pneumonia. However, whether XBJ could reduce the mortality of severe pneumonia patients is still controversial. In the past five years, more studies have been published on mortality, length of hospital stay, and duration of mechanical ventilation in severe pneumonia treated with XBJ. Therefore, this systematic review and meta-analysis, based on the currently published related randomized controlled trials (RCTs), is going to provide further evidence for clinical treatment of XBJ.

## 2. Materials and Methods

### 2.1. Registration

The registration number of PROSPERO is CRD42020173729.

### 2.2. Research Type

The research included blinded or nonblinded RCTs of XBJ combined with western medicine treatment. The language was limited to Chinese or English.

### 2.3. Research Objects

Adult patients with severe pneumonia were included; the diagnostic criteria must meet any of the following for severe pneumonia: (1) “Guidelines for the Diagnosis and Treatment of Community-Acquired Pneumonia in Chinese Adults” developed by the Respiratory Branch of Chinese Medical Association in 2016 [8]; (2) “Guidelines for Diagnosis and Treatment of Community-Acquired Pneumonia” made by the Respiratory Branch of Chinese Medical Association in 2006 [9]; (3) “Adult Community-Acquired Pneumonia (CAP) Guidelines for Diagnosis and Treatment” issued by the American Thoracic Society/American Society of Infectious Diseases (AST/IDSA) in 2007 [10]; (4) “Guidelines for the Management of Adults with Community-Acquired Pneumonia. Diagnosis, Assessment of Severity, Antimicrobial Therapy, and Prevention” formulated by the American Thoracic Society in 2001 [11].

### 2.4. Intervention Measures

The control group was given conventional western treatments such as anti-infectives, phlegm reduction medicines, mechanical ventilation, nutritional support, and so on. In the experimental group, adjuvant treatment was added with XBJ on the basis of conventional western treatment. In order to reduce the heterogeneity of the analysis of results, this study chose the literature with an intervention course of 7 days and observation index data on the 7th day.

### 2.5. Outcomes

One or more outcome indicators of the following must be involved: primary outcomes: (1) effective rate, (2) 28-day mortality; secondary outcomes: (1) length of intensive care unit (ICU) stay time, (2) duration of mechanical ventilation; (3) C-reactive protein (CRP), (4) procalcitonin (PCT), (5) white blood cells (WBC), (6) tumor necrosis factor-*α* (TNF-*α*), (7) interleukin-6 (IL-6), (8) D-dimer, (9) adverse reactions.

### 2.6. Exclusion Criteria

Exclusion criteria include the following:

1. Republished studies.
2. Unclear diagnostic criteria for severe pneumonia.
3. Nonadult studies.
4. Combination with other critically ill patients (tumor, pulmonary fibrosis, tuberculosis, secondary respiratory failure in other systems, etc.).
5. Combination with other proprietary Chinese medicines with effects of regulating blood coagulation or anti-inflammation.

### 2.7. Retrieval Strategy

The English databases of PubMed, Cochrane Library, Web of Science, and Embase, as well as the Chinese databases of CNKI, WanFang, and VIP, were retrieved to look up all the RCTs that used XBJ for severe pneumonia by the computer since the databases were established until February 2020. The search strategy for English databases was “pneumonia [Title/Abstract] AND Xuebijing [Title/Abstract].” The search strategy for Chinese databases was (“zhong zheng fei yan (han yu pin yin) [Title/Abstract]” OR “zhong zheng fei bu gan ran (han yu pin yin) [Title/Abstract]”) AND “xue bi jing (han yu pin yin) [Title/Abstract].”

### 2.8. Data Extraction

Two researchers read and screened the literature according to the inclusion and exclusion criteria back-to-back. After being extracted, the data was cross-checked. If there were differences, a third researcher joined the discussion. Extracted data included author, year of publication, region, sample size, age distribution, baseline characteristics, intervention dose, course of treatment, method of randomization, allocation concealment, blind method, data integrity, and outcomes.

### 2.9. Quality Assessment

The bias risk assessment tool for RCTs recommend by Cochrane Handbook 5.1.0 was used for quality evaluation, which included random sequence generation, allocation concealment, blinding method, incomplete outcome data, selective reporting, and other bias. Two researchers conducted a back-to-back bias risk assessment and then cross-checked the results. In case of disagreement, a third researcher joined the discussion.

### 2.10. Statistical Analysis

Meta-analysis was performed using RevMan 5.3 and Stata 16.0 statistical software. Outcomes were expressed as risk ratio (RR) for dichotomous variables and mean difference (MD) or standardized mean difference (SMD) for continuous variables together with 95% confidence intervals (CIs). Chi-square distribution (*X*^2^) *Q* test and *I*^2^ index were introduced to judge the heterogeneity of the research results. *P* > 0.1 and *I*^2^ <50% indicated that there was no statistical heterogeneity between the results and a fixed-effect model was used for meta-analysis. *P* ≤ 0.1 and *I*^2^ ≥ 50% indicated that there was statistical heterogeneity between the results and a random effect model was used for meta-analysis. If there was obvious clinical heterogeneity, subgroup meta-analysis or descriptive analysis was conducted. Inverted funnel chart and Egger's test were used to detect publication bias where there were more than 10 trials in the meta-analysis.

## 3. Results

### 3.1. Description of Included Studies

**Figure 1:**
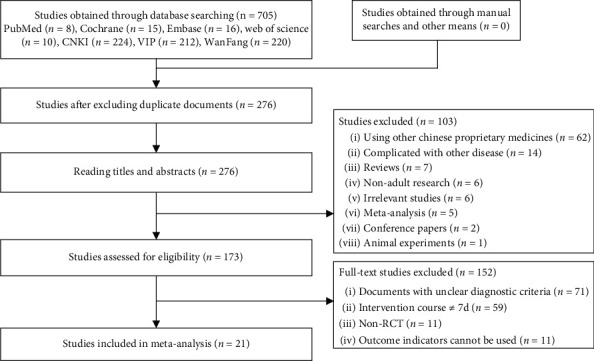
Flowchart of literature screening.

**Figure 2:**
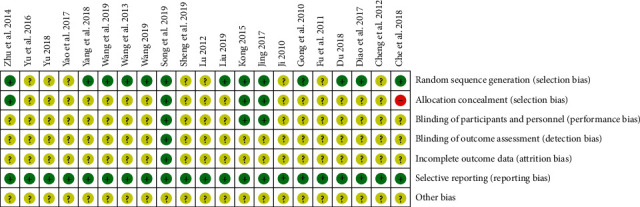
Evaluation of literature quality.

A total of 705 studies were obtained in the initial examination. After screening, 21 studies that met the criteria were finally included [12–32], involving 2072 patients, 1030 in the experimental group and 1042 in the control group. There were 20 papers [13–32] in Chinese and 1 paper [12] in English. There were two PhD theses. [13, 14] All research was conducted in China. The screening process is shown in Figure 1, and the risk of bias of the included trials is shown in Figure 2. The characteristics of the included studies are presented in Table 1.

### 3.2. Primary Outcomes

#### 3.2.1. Effective Rate

A total of 11 studies reported effective rate of treatment [14–24], and the evaluation criteria adopted by these studies were basically the same. Significantly effective rate was reported as follows: within 7 days, clinical symptoms such as cough, sputum, fever, and dyspnea were completely relieved, positive signs of lung examination disappeared, the shadow area of lungs on the X-ray was absorbed by more than 50%, laboratory examination was completely normal, and pathogen test turned negative in sputum specimens. Effective rate was reported as follows: within 7 days, the above clinical symptoms were partially relieved, signs of lung examination improved, shadow area of lungs on the X-ray absorbed was <50%, laboratory examination was significantly improved, and pathogen in sputum specimens was partially cleared or replaced. Invalid rate was reported as follows: within 7 days, no clinical symptoms were improved, positive signs of lung examination did not improve or worsen, the shadow area of lungs on the X-ray had no obvious absorption or had aggravation, there was no improvement or aggravation in the laboratory examination, and the examination of the sputum pathogen did not turn negative. Effective rate = (significantly effective cases + effective cases)/number of cases. According to the dosage of XBJ, studies were divided into two subgroups of 50 ml bid and 100 ml bid.

**Figure 3:**
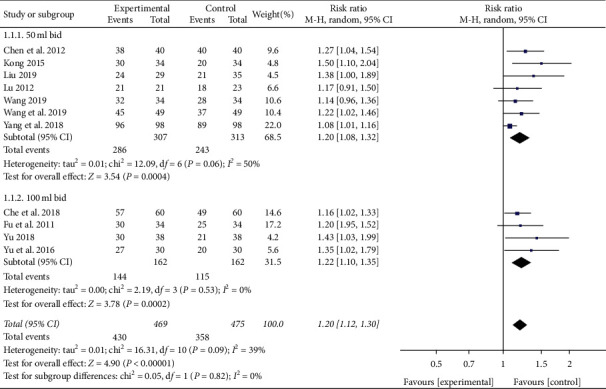
Effect of XBJ on effective rate in patients with severe pneumonia.

**Figure 4:**
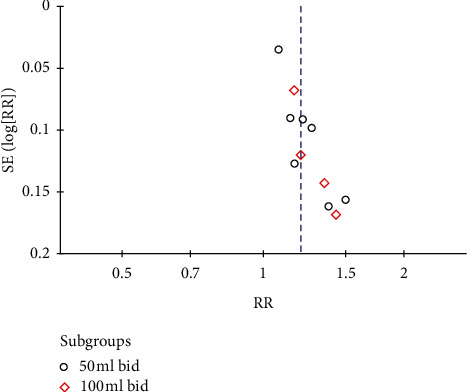
Funnel chart of the effectiveness of XBJ in treating severe pneumonia.

There were 7 studies in the 50 ml bid subgroup [14–20]. Heterogeneity was detected between these trials (*P* = 0.06, *I*^2^ = 50%), so random effect model was adopted. Meta-analysis results showed that the effective rate of XBJ group was higher than the control group (RR = 1.20, 95% CI [1.08, 1.32], *P* = 0.0004). There were 4 studies in the 100 ml bid subgroup [21–24]. No heterogeneity was detected between these trials (*P* = 0.53, *I*^2^ = 0%). Meta-analysis of random effect model result showed that the effective rate of XBJ group was higher than the control group (RR = 1.22, 95% CI [1.10, 1.35], *P* = 0.0002), as shown in Figure 3. Therefore, the results of the two subgroups showed that, regardless of whether the dose was 50 ml bid or 100 ml bid, the effective rate of the XBJ group was significantly higher than that of the control group, as shown in Figure 3.  Figure 4 shows the funnel chart of the treatment efficiency of the XBJ group and the control group. The symmetry is poor, and the data is to the right, indicating that there may be selectivity and publication bias. Egger's test showed that beta1 = 1.91, SE of beta1 = 0.601, *z* = 3.18, and Prob > |*z*| = 0.0015 (*P* = 0.0015), which also meant that there may be selectivity and publication bias.

#### 3.2.2. 28-Day Mortality

**Figure 5:**
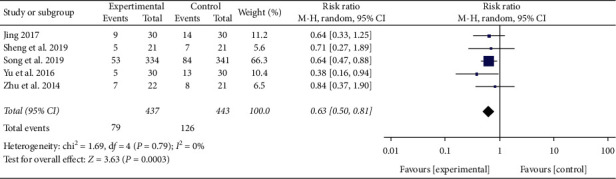
Effect of XBJ on 28-day mortality in patients with severe pneumonia.

A total of 5 studies reported a 28-day mortality rate [12, 13, 21, 25, 26]. No heterogeneity was detected between these trials (*P* = 0.79, *I*^2^ = 0%). Meta-analysis results of the fixed-effect model showed that the 28-day mortality in XBJ group was significantly lower than the control group (RR = 0.63, 95% CI [0.50, 0.81], *P* = 0.0003), as shown in Figure 5. Among them, the dose of XBJ used in four studies was 100 ml bid [12, 13, 21, 26], and only one study used 50 ml bid [25].

### 3.3. Secondary Outcomes

#### 3.3.1. ICU Stay Time

**Figure 6:**
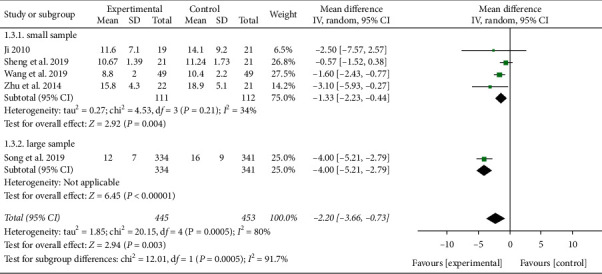
Effect of Xuebijing on ICU stay time in patients with severe pneumonia.

Five studies reported ICU stay time [12, 16, 25, 26, 28]. Heterogeneity was detected between these trials (*P* = 0.0005, *I*^2^ = 80%). The sample size of Song et al.'s study [12] was found to be significantly larger than that of other studies, and heterogeneity may have come from the difference of sample size. When subgroup analysis was carried out according to the sample size, the heterogeneity decreased significantly. Meta-analysis results showed that the ICU stay time of XBJ group was shorter than that of the control group (RR = −1.33, 95% CI [−2.23, −0.44], *P* = 0.004), as shown in Figure 6. Large sample size may better reflect the response of the population, so larger-sample research needs to be carried out.

#### 3.3.2. Duration of Mechanical Ventilation

**Figure 7:**
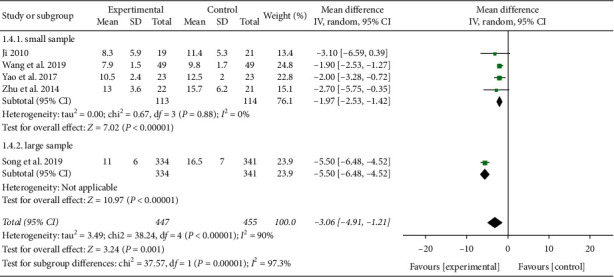
Effect of Xuebijing on mechanical ventilation time in patients with severe pneumonia.

A total of 5 studies reported duration of mechanical ventilation [12, 16, 26, 28, 29]. Heterogeneity was detected between these trials (*P* < 0.00001, *I*^2^ = 90%). The sample size of Song et al.'s study [12] was found significantly larger than that of other studies, and heterogeneity may have come from the difference of sample size. When subgroup analysis was carried out according to the sample size, the heterogeneity decreased significantly. Meta-analysis results showed that duration of mechanical ventilation of the XBJ group was shorter than that of the control group (RR = −1.97, 95% CI [−2.53, −1.42], *P* < 0.00001), as shown in Figure 7. Large sample size may better reflect the response of the population, so larger-sample research needs to be carried out.

#### 3.3.3. CRP

**Figure 8:**
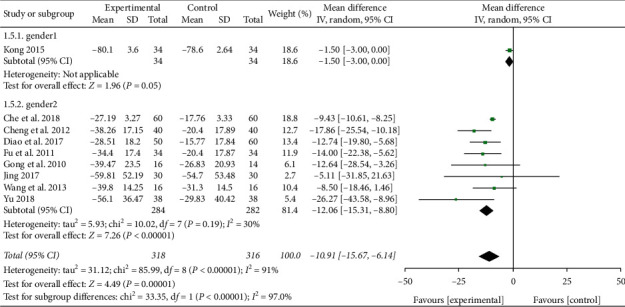
Effect of XBJ on the level of CRP in patients with severe pneumonia.

A total of 9 studies reported CRP level in plasma [13, 18, 19, 22–24, 27, 30, 31]. Heterogeneity was detected between these trials (*P* < 0.00001, *I*^2^ = 91%). Male proportion of subjects in Kong's study [18] was found to be significantly higher than that of other studies, and heterogeneity may have come from the difference in gender composition. When subgroup analysis was carried out according to the gender, the heterogeneity decreased significantly. Meta-analysis results showed that the CRP level of the XBJ group was lower than that of the control group (MD = −12.06, 95% CI [−15.31, −8.08], *P* < 0.00001), as shown in Figure 8. Therefore, when Xuebijing is used to treat severe pneumonia, gender may be an influencing factor in the change of CRP level.

#### 3.3.4. PCT

**Figure 9:**
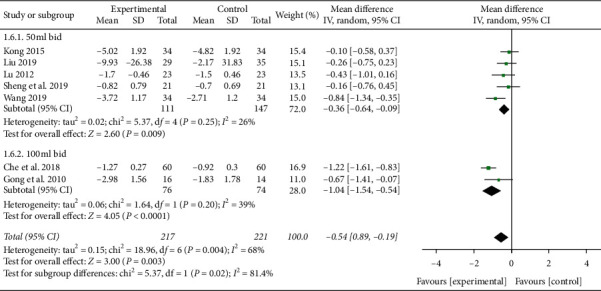
Effect of XBJ on the level of PCT in patients with severe pneumonia.

Seven studies reported PCT level in plasma [14, 15, 18, 20, 23, 25, 27]. Heterogeneity was detected between these trials (*P* = 0.004, *I*^2^ = 68%). When subgroup analysis was carried out according to the dose of XBJ, the heterogeneity decreased significantly. There were 5 studies in the 50 ml bid subgroup [14, 15, 18, 20, 25]. No heterogeneity was detected between these trials (*P* = 0.25, *I*^2^ = 26%). Meta-analysis results showed that the PCT level of the XBJ group was lower than that of the control group (MD = −0.36, 95% CI [−0.64, −0.09], *P* = 0.009). There were 2 studies in the 100 ml bid subgroup [23, 27]. No heterogeneity was detected between the two trials (*P* = 0.20, *I*^2^ = 39%). Meta-analysis results showed that the PCT level of the XBJ group was lower than that of the control group (MD = −1.04, 95% CI [−1.54, −0.54], *P* < 0.0001). Therefore, the results of the two subgroups showed that, regardless of whether the dose was 50 ml bid or 100 ml bid, the PCT level of the XBJ group was lower than that of the control group, as shown in Figure 9. Accordingly, when Xuebijing is used to treat severe pneumonia, the dose of XBJ may be an influencing factor in the change of PCT level.

#### 3.3.5. WBC

**Figure 10:**
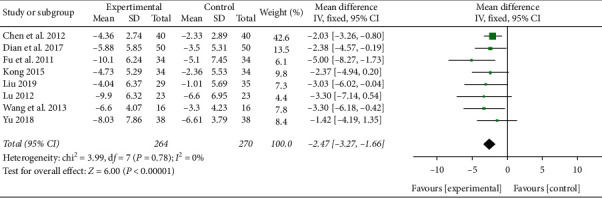
Effect of XBJ on the level of WBC in patients with severe pneumonia.

A total of 8 studies reported WBC level in plasma [14, 18–20, 22, 24, 30, 31]. No heterogeneity was detected between these trials (*P* = 0.78, *I*^2^ = 0%). Meta-analysis results of the fixed-effect model showed that the level of WBC in the XBJ group was significantly lower than that of the control group (MD = −2.47, 95% CI [−3.27, −1.66], *P* < 0.00001), as shown in Figure 10.

#### 3.3.6. TNF-*α*

Six studies reported the serum TNF-*α* level [14, 21, 27, 29, 30, 32]. Heterogeneity was detected between these trials (*P* < 0.00001, *I*^2^ = 86%). Heterogeneity between the six studies which cannot be reduced by subgroup analysis may be due to the influence of multiple factors such as sample size, age and gender composition, and treatment dose. Hence, descriptive analysis was conducted. The 6 studies, respectively, showed that the TNF-*α* level of the XBJ group was lower compared with the control group with statistical significance indicating that XBJ combined with basic treatment could reduce the level of TNF-*α* in patients with severe pneumonia.

#### 3.3.7. IL-6

A total of 4 studies have reported the serum IL-6 level [12, 14, 29, 30]. Heterogeneity was detected between these trials (*P* < 0.00001, *I*^2^ = 91%). Heterogeneity between the four studies which cannot be reduced by subgroup analysis may be due to the simultaneous influence of multiple factors such as sample size, age and gender composition, and treatment dose. Hence, descriptive analysis was conducted. The four studies, respectively, showed that the IL-6 level of the XBJ group was lower compared with the control group with statistical significance indicating that XBJ combined with basic treatment could reduce the level of IL-6 in patients with severe pneumonia.

#### 3.3.8. D-Dimer

**Figure 11:**
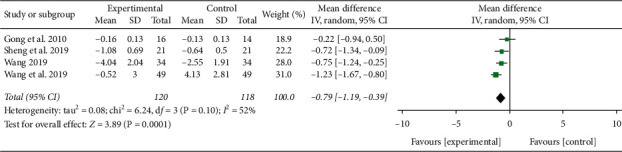
Effect of XBJ on the level of D-dimer in patients with severe pneumonia.

Four studies reported the serum D-dimer level [15, 16, 25, 27]. Heterogeneity was detected between these trials (*P* < 0.10, *I*^2^ = 52%), so random effect model was adopted. Meta-analysis result showed that the level of D-dimer was lower compared with the control group (SMD = −0.79, 95% CI [−1.19, −0.39], *P* = 0.0001), as shown in Figure 11. In addition, Liu Xinyan's study found that the platelet level of the XBJ group was significantly lower than that of the control group after treatment [14]. The study of Gong et al. found that the time of thromboplastin in the XBJ group was significantly shorter than that of the control group after treatment [27].

#### 3.3.9. Adverse Reactions

**Figure 12:**
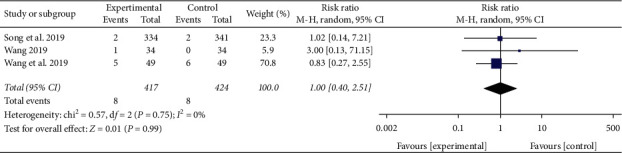
Meta-analysis of adverse reactions of XBJ in patients with severe pneumonia.

A total of 5 studies reported adverse reactions [12, 13, 15, 16, 20], of which Jing [13] and Lu [20] did not find any adverse reactions. No heterogeneity was detected between these trials (*P* = 0.75, *I*^2^ = 0%). Meta-analysis results of the fixed-effect model showed that there was no significant difference in adverse reactions between the two groups (RR = 1, 95% CI [0.40, 2.51], *P* = 0.99), as shown in Figure 12.

## 4. Discussion

### 4.1. The Main Findings of This Study Compared to Previous Studies

Compared with the previous meta-analyses, the quality of the included literature has improved. All the studies included in this paper used 7-day intervention courses. The drug instructions for Xuebijing do not clearly stipulate the course of treatment. In clinical practice, the course of treatment is mostly 7 days, sometimes extended to 14 days. However, in the evaluation of efficacy, the observation time is usually 7 days, so studies of 7-day treatment courses containing observation data on the 7th day were included. Thus, heterogeneity of the study results could be controlled. This study shows that XBJ combined with conventional western treatment of severe pneumonia is effective and could reduce the level of inflammation reaction, which is consistent with the results of previous meta-analyses [5–7].

However, whether XBJ could reduce the mortality of patients with severe pneumonia has been controversial. Our study found that XBJ in the treatment of severe pneumonia could improve 28-day mortality of patients. However, meta-analyses conducted by Bai et al. [5] and Zhu et al. [6] both reported that there was no difference in the mortality between XBJ group and control group in treating severe pneumonia. There are two main reasons for their results. On the one hand, the mortality was observed only during the patient's hospitalization but was not followed up after the patients were discharged. It is speculated that XBJ must still have a therapeutic effect after the course of treatment. On the other hand, this may be related to the dosage of XBJ. In Zhu's study, three articles reported mortality, and the dosage of XBJ used was 50 ml bid. In Bai's study, two articles reported mortality; one article used 50 ml bid and the other used 100 ml bid. The dosage of XBJ used in both studies was lower compared with this research.

In this research, 5 articles reported 28-day mortality [12, 13, 21, 25, 26], four of which used XBJ at a dose of 100 ml bid [12, 13, 21, 26], and only one used XBJ at a dose of 50 ml bid [25]. In addition, Wang et al. (50 ml bid) [16], Liu (50 ml bid) [14], and Gong et al. (100 ml bid) [27] reported that the mortality was not statistically different from that of the control group. Similarly, they observed the mortality only during the patient's hospitalization, but did not follow up to observe the 28-day mortality. Simultaneously, dosage of XBJ used in their studies was 50 ml bid, 50 ml bid, and 100 ml bid, respectively. Therefore, this research shows that XBJ may have a delayed treatment effect, and in order to reduce the 28-day mortality rate of patients with severe pneumonia, the recommended dose of XBJ should be 100 ml bid for at least 7 days.

Different from previous studies, this research also conducted a meta-analysis of ICU stay time, duration of mechanical ventilation, and adverse reactions of patients with severe pneumonia, finding that XBJ could shorten the ICU stay time and the duration of mechanical ventilation and have no increased adverse reactions. This provides a further evidence-based basis for the clinical use of XBJ in the treatment of severe pneumonia.

### 4.2. Other Findings

This study also found that XBJ could shorten thromboplastin time, reduce levels of serum D-dimer and platelets, and correct coagulopathy of patients [14, 16, 25, 27]. Many studies have found that, in severe infections, the inflammatory factors in patients could activate blood coagulation factors and start the blood coagulation process, leading to the body's microcirculation disorders, thereby exacerbating the occurrence of multiple organ failure [33, 34]. This has been recorded a long time ago in the ancient Chinese medicine books. “WenYiLun” says “Evil heat has long been restrained, but there is no way to vent, so it stayed in the meridians and became stasis.” “YiLinGaiCuo” states that “poison burnt its blood, blood was burnt, and its blood must coagulate.” They explain the relationship between inflammation and coagulation [35].

Xuebijing, an intravenous preparation, was approved by the China Food and Drug Administration (China FDA) in 2004. Xuebijing is prepared from a combination of *Carthamus tinctorius* flowers (Honghua in Chinese), *Paeonia lactiflora* roots (Chishao), *Ligusticum chuanxiong* rhizomes (Chuanxiong), *Salvia miltiorrhiza* roots (Danshen), and *Angelica sinensis* roots (Danggui). It has the functions of anti-inflammation, antioxidation, improving blood coagulation, improving microcirculation, regulating immune function, etc. [36]. The mechanism of XBJ in the treatment of severe pneumonia may be to improve the patient's microcirculation and organ function by suppressing the excessive inflammatory response and correcting the coagulation disorder.

### 4.3. Defects and Deficiencies

Some of the literature included in this study is of low quality. In addition, there are still some clinical trials with small sample size and inadequate design. All of the studies were conducted in China. The subjects were basically Chinese. Larger-sample size, multiregion, and multicenter clinical RCTs are required to verify whether XBJ has the same effect on people in different regions or people of different ethnicities.

## Figures and Tables

**Table 1:** Characteristics of included studies.

Study ID	Sample size	Gender (men%)	Mean age (years)	Diagnostic criteria	Randomization method	Blinding method	Intervention		Course (d)	Follow-up time (d)	Outcome indicators
	T/C	T/C	T/C				C	T			
Song et al. [12]	334/341	67.1/68.6	58.7/58.1	America 2007 [10]	Interactive web response system	Double blinded	BT	BT + XBJ 100 ml bid	7	28	2, 3, 4, 11
Jing [13]	30/30	60.0/66.7	61.5/62.1	America 2007	Allocation sequence, assigned random number by system	Double blinded	BT	BT + XBJ 100 ml bid	7	28	2, 5, 11
Liu [14]	29/35	65.5/60.0	71.8/77.7	China 2016 [8]	Random number table	Unstated	BT	BT + XBJ 50 ml bid	7	—	1, 6, 7, 8, 9
Wang [15]	34/34	64.7/58.8	75.2/75.6	China 2016	Random number table	Unstated	BT (Biapenem 0.3 g q8 h)	BT + XBJ 50 ml bid	7	—	1, 6, 11
Wang et al. 2019 [16]	49/49	57.1/61.2	54.8/53.3	China 2016	Draw method	Unstated	BT (Cefoperazone sulbactam 3.0 g q12 h + Levofloxacin 0.5 g qd)	BT + XBJ 50 ml bid	7	—	1, 3, 4, 11
Yang and Xu [17]	98/98	63.3/66.3	57.4/53.6	America 2007	Random number table	Unstated	BT	BT + XBJ 50 ml bid	7	—	1
Kong [18]	34/34	82.4/85.3	48.9/50.6	America 2007	Interactive web response system	Double blinded	BT	BT + XBJ 50 ml bid	7	—	1, 5, 6, 7
Cheng et al. [19]	40/40	62.5/52.5	—	America 2001 [11]	Unstated	Unstated	BT	BT + XBJ 50 ml bid	7	—	1, 5, 7
Lu [20]	23/23	65.2/60.9	71.04/69.3	America 2001	Unstated	Unstated	BT	BT + XBJ 50 ml bid	7	—	1, 6, 7, 11
Yu and Ma [21]	30/30	76.7/70.0	52.1/54.5	China 2006 [9]	Unstated	Unstated	BT	BT + XBJ 100 ml bid	7	28	1, 2, 8, 9
Yu [22]	38/38	57.9/52.6	65.9/67.1	America 2007	Unstated	Unstated	BT	BT + XBJ 100 ml bid	7	—	1, 5, 7
Che et al. [23]	60/60	—	—	America 2007	Random number table	Unstated	BT	BT + XBJ 100 ml bid	7	—	1, 5, 6
Fu and Ge [24]	34/34	70.6/64.7	69.6/67.8	America 2001	Unstated	Unstated	BT	BT + XBJ 100 ml bid	7	—	1, 5, 7
Sheng et al. [25]	21/21	81.0/61.9	43.6/44.3	China 2016	Unstated	Unstated	BT	BT + XBJ 50 ml bid	7	28	2, 3, 6
Zhu and Liu [26]	22/21	72.7/66.7	68.3/64.6	America 2007	Random number table	Unstated	BT	BT + XBJ 100 ml bid	7	28	2, 3, 4
Gong et al. [27]	16/14	56.3/78.6	57.0/59.0	China 2006	Random number table	Unstated	BT	BT + XBJ 100 ml bid	7	—	5, 6, 8
Ji [28]	19/21	—	—	China 2006	Unstated	Unstated	BT	BT + XBJ 100 ml bid	7	—	3, 4
Yao et al. [29]	23/23	73.9/65.2	45.6/44.8	America 2007	Unstated	Unstated	BT	BT + XBJ 100 ml bid	7	—	4, 8, 9
Diao et al. [30]	50/50	—	—	America 2007	Random number table	Unstated	BT	BT + XBJ 50 ml bid	7	—	5, 7, 8, 9
Wang et al. [31]	16/16	—	—	America 2001	Random number table	Unstated	BT	BT + XBJ 50 ml bid	7	—	5, 7
Du [32]	30/30	60.0/53.3	58.6/57.9	America 2007	Random number table	Unstated	BT	BT + XBJ 50 ml bid	7	—	8

*Note.* T: intervention group; C: control group; BT: basic treatment including rational antibiotic treatment, early fluid resuscitation, mechanical ventilation, use of glucocorticoids, nutrition support, maintenance of homeostatic equilibrium, and other comprehensive therapies. Outcome indicators: 1, effective rate; 2, 28-day mortality; 3, ICU stay time; 4, duration of mechanical ventilation; 5, CRP; 6, PCT; 7, WBC; 8, TNF-*α*; 9, IL-6; 10, D-dimer; 11, adverse reactions.

## Data Availability

The data used to support the findings of this study are included within the article.

## Abbreviations

XBJ: Xuebijing injection
RCTs: Randomized controlled trials
RRs: Risk ratios
CIs: Confidence intervals
CNKI: China National Knowledge Infrastructure
ICU: Intensive care unit
CRP: C-reactive protein
PCT: Procalcitonin
WBC: White blood cell
TNF: Tumor necrosis factor
IL: Interleukin.

## References

[1] Chalmers J. D., Mandal P., Singanayagam A. (2011). Severity assessment tools to guide ICU admission in community-acquired pneumonia: systematic review and meta-analysis. *Intensive Care Medicine*.

[2] Ryan D., Connolly R., Fennell J. (2014). Aetiology of community-acquired pneumonia in the ICU setting and its effect on mortality, length of mechanical ventilation and length of ICU stay: a 1-year retrospective review. *Critical Care*.

[3] Sopena N., Heras E., Casas I. (2014). Risk factors for hospital-acquired pneumonia outside the intensive care unit: a case-control study. *American Journal of Infection Control*.

[4] Ruuskanen O., Lahti E., Jennings L. C., Murdoch D. R. (2011). Viral pneumonia. *The Lancet*.

[5] Bai Y. P., Wang H. F., Wang M. H. (2012). Systematic review of randomized controlled trials of adjuvant therapy of traditional Chinese medicine compound Xuebijing injection in the treatment of severe pneumonia. *Chinese Journal of Integrated Traditional and Western Medicine*.

[6] Zhu M. J., Zhang G., Hu M. H. (2014). A systematic review of the efficacy of Huayujingdu Xuebijing injection in the treatment of severe pneumonia. *Chinese Journal of Evidence-Based Medicine*.

[7] Feng B., Wang P., Ning Z. (2015). Meta-analysis of a randomized controlled study of Xuebijing injection in adjuvant treatment of severe pneumonia. *Tianjin Pharmaceuticals*.

[8] Respiratory Branch of Chinese Medical Association (2016). Guidelines for the diagnosis and treatment of Chinese community-acquired pneumonia (2016 edition). *Chinese Journal of Tuberculosis and Respiratory Diseases*.

[9] Respiratory Branch of Chinese Medical Association (2006). Guidelines for the diagnosis and treatment of community-acquired pneumonia. *Chinese Journal of Tuberculosis and Respiratory Diseases*.

[10] Mandell L. A., Wunderink R. G., Anzueto A. (2007). Infectious Diseases Society of America/American Thoracic Society Consensus guidelines on the management of community-acquired pneumonia in adults. *Clinical Infectious Diseases*.

[11] Niederman M. S., Mandell L. A., Anzueto A. (2001). Guidelines for the management of adults with community-acquired pneumonia. Diagnosis, assessment of severity, antimicrobial therapy, and prevention. *American Journal of Respiratory and Critical Care Medicine*.

[12] Song Y., Yao C., Yao Y. (2019). XueBiJing injection versus placebo for critically ill patients with severe community-acquired pneumonia: a randomized controlled trial. *Critical Care Medicine*.

[13] Jing S. S. (2017). *Clinical Observation of Xuebijing Injection in Treating Severe Pneumonia-Induced Sepsis (Stagnation of Blood Stasis and Toxin Mutual Syndrome)*.

[14] Liu X. Y. (2019). *Research on the Clinical Effect of Xuebijing Injection on Severe Pneumonia Based on the Theory of Lung Network*.

[15] Wang Z. W. (2019). Efficacy evaluation of Biapenem combined with Xuebijing injection in the treatment of acquired pneumonia in senile critical hospitals. *Hubei Journal of Traditional Chinese Medicine*.

[16] Wang D. L., Zhao L. D., Li L. J. (2019). Effect of Xuebijing combined with cefoperazone sulbactam sodium and levofloxacin on immune function, coagulation function and curative effect in patients with severe community-acquired pneumonia. *Chinese Journal of Hospital Pharmacy*.

[17] Yang D. M., Xu D. F. (2018). The effect of Xuebijing adjuvant treatment on severe pneumonia and its effect on renal function and serum Th1/Th2 cytokines. *Drug Evaluation Research*.

[18] Kong L. Y. (2015). Analysis of clinical efficacy of Xuebijing injection in the treatment of severe community-acquired pneumonia in ICU. *Tianjin University of Traditional Chinese Medicine*.

[19] Cheng X. M., Chen X. Y., Shen W. (2012). Clinical study of Xuebijing combined with antibiotics in the treatment of severe pneumonia. *China Health Nutrition (Late Journal)*.

[20] Lu W. X. (2012). Clinical experience of combined treatment of Chinese and Western medicine in the treatment of elderly severe community-acquired pneumonia. *Inner Mongolian Traditional Chinese Medicine*.

[21] Yu H. C., Ma Y. D., Yan S. (2016). Observation of the effect of Xuebijing on severe pneumonia and its influence on inflammatory factors, oxidation and antioxidant factors. *China Practical Medicine*.

[22] Yu Y. (2018). Clinical observation on 76 cases of severe pneumonia treated with Chinese and western medicine. *Chinese Journal of Metallurgical Industry Medicine*.

[23] Che X. Y., Chen B., Song Y. (2018). Effects of Xuebijing combined with antibacterial drugs on serum infection indexes, acute proteins and stress hormones in patients with severe ICU pneumonia. *Practical Drugs and Clinical Medicine*.

[24] Fu Y. H., Ge G. P. (2011). Observation of curative effect of Xuebijing injection combined with conventional Western medicine therapy on severe pneumonia in the elderly. *Shanghai Journal of Traditional Chinese Medicine*.

[25] Sheng N., Zhang L. L., Lu C. F. (2019). Clinical efficacy of Xuebijing in severe pneumonia. *Journal of Tropical Medicine*.

[26] Zhu J. J., Liu J. (2014). The effect of Xuebijing on inflammatory cytokines in serum and bronchoalveolar lavage fluid of patients with severe pneumonia. *Jiangsu Medicine*.

[27] Gong B. L., Zhang Y., Xu Q. X. (2010). Changes of nuclear transcription factor-*κ*B DNA binding activity in patients with severe pneumonia and the intervention effect of Xuebijing injection. *China Critical Care Medicine*.

[28] Ji M. X. (2010). Clinical observation of Xuebijing injection on changes of peripheral blood T lymphocytes in patients with severe pneumonia. *Strait Pharmaceuticals*.

[29] Yao L., Liu Y. N., Hou G. (2017). Study on the regulation of Xuebijing injection on the body’s immune function in severe pneumonia. *Journal of Hubei University of Traditional Chinese Medicine*.

[30] Diao Y. F., Zhang S. J., Zhao W. Y. (2017). Effect of Xuebijing injection on plasma IL-6 and TNF-*α* levels in patients with severe pneumonia. *Chinese Herbal Medicine*.

[31] Wang Z. G., Long Y. Z., Zhang G. M. (2013). Observation on the therapeutic effect of Xuebijing on elderly patients with severe pneumonia. *Jiangxi Medicine*.

[32] Du L. (2018). Observation of the efficacy of Xuebijing injection in the treatment of severe pneumonia combined with ARDS. *Frontiers of Medicine*.

[33] Ding Z., Gu X. Y. (2019). Discussion on the relationship between coagulopathy and the severity of sepsis in patients with ICU infection. *Chinese and Foreign Medical*.

[34] Song L. C., Han Z. H. (2017). Research progress of sepsis-related coagulation dysfunction mechanism and treatment. *Chinese Journal of Critical Care Medicine (Electronic Version)*.

[35] Tang S. H., Jiang W. M. (2014). The formation and clinical significance of the pathogenesis theory of “stasis of heat”-One of the relevant academic experiences of Z.Y.Zhou, a master of traditional Chinese medicine stasis of heat. *Jiangsu Chinese Medicine*.

[36] Jing S. S., Chen X. T., Liu Z. (2017). Research progress on the mechanism of Xuebijing in the treatment of severe pneumonia. *China Modern Drug Application*.

